# The Medical Schools of the United Kingdom

**Published:** 1921-08-27

**Authors:** 


### THE MEDICAL STUDENT AND HIS WORK.

### THE MEDICAL SCHOOLS OF THE UNITED KINGDOM.

### LONDON.

**University of London, University College** (Faculty of Medical Sciences) : University Centre for Preliminary and Intermediate Medical Studies.—The College Faculty of Medical Sciences comprises the departments of physics, chemistry, botany, and zoology (the preliminary medical sciences), also the departments of anatomy, physiology, and pharmacology (the intermediate medical sciences), and the department of hygiene and public health (post-graduate study). Composition Fees : For the courses required by the University of London : (1) For the medical course, 35 guineas. (2) For the second medical course, 80 guineas, payable in two instalments of 40 guineas each. For the medical education required by the Examination Board in England and the Society of Apothecaries : First examination, Parts I., II., III., 35 guineas. First examination, Part IV., and second examination, 80 guineas, payable in two instalments of 40 guineas each. The privileges of the Union Society (including the use of the gymnasium and the athletic ground at Perivale) are available to students. All departments in the Faculty of Medical Sciences are open to women students on the same terms as to men.

**Charing Cross Hospital.**—This hospital is fully organised for University teaching, and contains 300 beds. The courses of study are specially designed to meet the requirements of the University of London degrees, the Conjoint Board diplomas, and the final studies of the Universities of Oxford and Cambridge. Men and women students are admitted on equal terms. Fully equipped laboratories are available for pathological and bacteriological work. Fees: Entrance fees—(1) Primary and Intermediate, 10 guineas; (2) Final, 8 guineas. Annual fee (including Club and Library subscriptions), 26 guineas. The usual resident and students' appointments are open to students, and valuable prizes are awarded annually. Full information respecting the Medical School, and the course of study recommended, may be obtained on application to the Dean, W. J. Fenton, M.D., F.R.C.P., Charing Cross Hospital Medical School, London, W.C. 2.

**Guy's Hospital Medical School.**—The hospital, which is situated two minutes' walk from London Bridge, contains 644 beds. Students are appointed to dresserships and clerkships in the wards and out-patient departments on the sixteenth day of October, January, April, and July. Since 1904 the medical school buildings have been greatly extended. The Wills' Library was presented in 1903, the Gordon Museum in 1905. There is a Students' Club, and adjoining the Club are the Pavy Gymnasium, a covered swimming-bath, and a squash racquet court. The College, within which are the premises of the Students' Club, affords residential accommodation for about sixty students. There is an athletic ground of nine acres, situated at Honor Oak Park, distant about fifteen minutes by train from London Bridge. Fees and Courses.—*First Year.*—For Preliminary Science Course : £22 8s. for twelve months or less period, deducted from the entrance-fee, payable as a second year's student. A special fee of £7 is charged for materials for this course. *Second or Third Year* (after first M.B.) : Entrance-fee, £28. Annual composition-fee, £49, including all necessary materials. *Fourth Year* (after second M.B.) : Entrance-fee, £14. Annual composition-fee, £49, including all necessary materials. Provided a student has paid three annual composition-fees, a proportionate rebate will be allowed from the last on his obtaining an approved qualification at any time within nine months of the last payment. Entrance scholarships to the value of £660 awarded annually in September. For further particulars, and permission to be conducted over the school buildings, application should be made to the Dean, Guy's Hospital, S.E. 1.

**Guy's Hospital Dental School.**—The present buildings were extended and rebuilt in 1912. They now form a block of buildings occupying four sides of a courtyard and consisting of three floors. The whole professional training of the dental student is carried out at Guy's. Beds are provided in the wards of the hospital for patients suffering from fractures and diseases of the jaw, and weekly demonstrations are given on surgical cases of interest to dental students. For club and residential facilities see Guy's Medical School (above). The following appointments are open to students every year without examination : Eight house surgeons, twelve assistant house surgeons, twelve clinical assistants, ten assistant demonstrators. *Composition-fees* : Dental studentship, fifty guineas plus 33⅓ per cent. for each of the four years; dental and medical studentship, fifty guineas a year plus 33⅓ per cent. for the first three years; sixty guineas plus 33⅓ per cent. for the fourth; and forty guineas each plus 33⅓ per cent. for the fifth and six.

**King's College Hospital Medical School (University of London).**—King's College Hospital is situated at Denmark Hill, London, S.E. 5, and at present contains 400 beds. There are about 4,000 attendances per week in the casualty and out-patient departments. The hospital possesses every modern requirement for both patients and students. There is ample opportunity for any student who wishes to specialise in a subject to continue his studies with the help of junior staff appointments. The teaching of the preliminary subjects is given at King's College in the Strand, students coming to the hospital for their advanced work. Fifteen resident appointments are made half-yearly. The composition fee for advanced medical studies is 93 guineas if paid in one instalment, or 95 guineas if paid in two instalments, in addition to the entrance fee of 10 guineas. The composition fee for the whole University or Conjoint course is 200 guineas. Two Entrance Scholarships (£50 each), for University students, will be offered for competition in September 1921. Full particulars of these scholarships, and the school calendar, may be obtained by application to either of the following : H. Willoughby Lyle, M.D., B.S. (Lond.), F.R.C.S., *Dean* ; S. C. Ranner, M.A. (Cantab.), Secretary of the Medical School, King's College Hospital, Denmark Hill, London, S.E. 5.

**London Hospital Medical College.**—The London Hospital is the largest institution of its kind in England, and contains 950 beds. During last year 21,639 in-patients and 120,886 out-patients received treatment, while 7,804 major operations were performed. The clinical units in medicine and surgery co-ordinate the teaching of their subjects in the hospital. The directors and their assistants give practical instruction in elementary clinical medicine and surgery to all students before they are allowed to enter the wards and out-patient department. Senior students are encouraged to work with the units. Holders of resident appointments have free board. Special classes are held for the higher examinations of the University of London, the Royal College of Physicians, and the Royal College of Surgeons. Special entries for medical and surgical practice can be made. A residential hostel on hospital grounds is provided for the convenience of students. The Clubs' Union Athletic Ground of over thirteen acres is within easy reach of the hospital. Scholarships and Prizes : Price Scholarship £100, and one Entrance Scholarship £50, subjects of First Medical Examination at the University of London; Epsom College Scholarship, free education, subjects of the First Medical Examination as above; Price Scholarship, open to students of Oxford and Cambridge Universities, £52 10s., human anatomy and physiology. Numerous prizes are offered in the various subjects of the curriculum. Medical Research Funds provide valuable scholarships for men wishing to undertake research or desirous of preparing theses for University degrees. Dean : Professor William Wright, London Hospital Medical College, E. 1.

**London Hospital Dental School.** — The school, which is fully equipped on the most modern lines and with the latest appliances, is an integral part of the college and hospital. A systematic course of instruction in dental prosthesis is arranged for pupils. The laboratory is in charge of a skilled curator and his assistants. Students in the school have the advantage of pursuing the medical portion of the dental curriculum in a college and hospital, of which the Dental School is part. Lecturers : E. Sprawson, M.C., H. Chapman, H. D. Clapham, A. Dinnis, F. Butler, Professor William Wright, Professor H. E. Roaf, Dr. Paul Fildes, R. J. Probyn-Williams, J. D. Lyle, H. Candy; Director of Dental Studies : E. Sprawson, M.C., M.R.C.S., L.R.C.P., L.D.S.; Dean : Professor Wm. Wright, M.B., D.Sc., F.R.C.S.; Secretary : E. J. Burdon.

**London (Royal Free Hospital) School of Medicine for Women.**—The Royal Free Hospital is in Gray's Inn Road, and the London School of Medicine for Women is in Hunter Street, Brunswick Square, W.C. Under agreements the clinical practice of St. Mary's Hospital, the Elizabeth Garrett Anderson Hospital, the Cancer Hospital, the National Hospital for Nervous Diseases, and the Hospital for Sick Children are also available for students of the school. Residence is provided in the Students' Chambers attached to the school. There are numerous scholarships and prizes offered annually in connection with the school, among which may be mentioned the Isabel Thorne Scholarship of £30, the St. Dunstan's Medical Exhibition of £60 a year for three or five years, the Mabel Sharman-Crawford Scholarship of £20 a year for four years, the Mrs. George M. Smith Scholarship of £50 a year for three or five years, the Sir Owen Roberts Scholarship of £75 a year for four years, the Dr. Margaret Todd Scholarship of £37 10s. a year for four years, the Sarah Holborn Scholarship of £20 a year for three or five years, and the Agnes Guthrie Bursary for dental students of £50. Numerous resident appointments are available for students of the school after qualification. The session will begin on Monday, October 3.

**Middlesex Hospital.**—The hospital and medical school are situated in Mortimer Street, and only a few minutes' walk from Oxford Circus. The hospital contains 455 beds. The cancer wing (containing ninety-two beds) and special investigation laboratories offer unrivalled opportunities for the study of cancer, both in its clinical and pathological aspects. There are also electro-therapeutic and electro-cardiographic departments. The Bland-Sutton Institute of Pathology contains a lecture theatre, large laboratories for pathological, bacteriological, and clinical investigation, and smaller rooms for original research work. Special courses of instruction are arranged twice yearly for the Diploma of Public Health. Six house physicians, eight house surgeons, two obstetric and gynæcological surgeons, two casualty medical officers, two casualty surgical officers, one resident anæsthetist, and two resident officers to the special departments are appointed annually, without extra fee of any kind. Non-resident qualified clinical assistants are appointed to assist in the various out-patient departments. Numerous scholarships, medals, and prizes, to the value of over £1,000, are open for competition among students of the school. All students join the Amalgamated Students' Club, which includes the following : Cricket and Football Clubs, Athletic Clubs, a Musical Society, and a Chess Club. The winter session will open on Tuesday, October 4, at 3 p.m. For prospectus and full particulars application should be made to the Dean of the Medical School.

**St. Bartholomew's Hospital Medical School.**—St. Bartholomew's is the oldest and second largest hospital in London. The medical school is complete in every department, and provides lectures and practical laboratory teaching in the preliminary sciences and intermediate subjects, as well as in the purely professional and clinical parts of the curriculum. Scholarships and prizes to the total value of nearly £1,000 are awarded annually.

**St. George's Hospital.**—The hospital contains 350 beds, and has a convalescent hospital of 100 beds at Wimbledon. The usual resident and students' appointments are open to students. Scholarships and endowed prizes, amounting to £576, are awarded every year. The entire teaching and laboratories are now devoted to purely clinical subjects, to the great advantage of students in their fourth and fifth years of study. Arrangements have been made with the University of London for students who enter St. George's during the first, second, or third year of the curriculum to carry out the necessary courses of instruction at either University College or King's College. Students have therefore the advantages of the lectures and practical classes of these Colleges of the University during the preliminary and intermediate portions of their studies, and then complete their course in a school entirely devoted to medicine, surgery, and the study of disease. The St. George's Hospital Club has its athletic ground of 13½ acres on the Atkinson-Morley Hospital estate at Wimbledon. It provides a football and hockey ground and six tennis courts. Further particulars may be obtained on application to the Dean of the Medical School, Mr. R. R. James, F.R.C.S.

**St. Mary's Hospital Medical School.**—This Hospital and Medical School are situated close to Paddington Station (G.W.R.). It contains 305 beds, and by a scheme of affiliation, for teaching purposes, of the Paddington Infirmary, Paddington Green Children's Hospital, and Maida Vale Hospital for Nervous Diseases, the teaching facilities extend over 1,000 beds. The athletic ground (10 acres) is situated at Wembley. Clinical units in medicine and surgery were established in 1920, and have now been formally recognised by the University Grants Committee, St. Mary's being one of the six medical schools in London which enjoy this privilege. Lying-in beds have been recently added, and provide special facilities for the teaching of practical midwifery. There is an Institute of Pathology and Research, comprising seven special departments, the whole being under the personal direction of Sir Almroth Wright, F.R.S. Three Research Scholarships of £200 each are awarded annually to students working in the departments of the institute, and preparation is being made to open thirteen research beds. Clerkships in pathology and bacteriology, lasting for a period of three months, are open to students of the fifth year, and enable them to carry out the pathological and bacteriological investigations of the wards and learn the necessary technique under supervision. Sixty-four of these posts are available annually. The Medical School provides complete courses of instruction, and students can join at once on passing a preliminary examination in arts. Terms begin in October, January, and April. Five scholarships (value £100, £52 10s., £52 10s., £50, and £25) are awarded annually by competitive examination in September. Fees: Composition fee for entire curriculum (5½ years), £200 in one sum, or £210 by five annual instalments; for clinical curriculum (2½ years), 90 guineas in one sum, or 95 guineas by two annual instalments. As an alternative, students may pay an annual fee of 40 guineas, with an entrance fee of 10 guineas.

**St. Thomas's Hospital.**—The hospital occupies a unique position by the river, and contains 630 beds. The in-patients last year numbered 8,362, whilst the number of attendances as out-patients was 379,329. The tuberculosis department forms a part of the Lambeth scheme for treatment of patients and instruction. The venereal department has been established as part of the L.C.C. scheme. There is a speech clinic in connection with the children's department. Clinical teaching in the wards, out-patient, and special departments is available every day of the week. All appointments in the hospital are open to students without extra fee. The Students' Club comprises a spacious restaurant, smoking and reading rooms, and there is no occasion for students to leave the hospital buildings during working hours. The annual fees are £25 for the first year (preliminary subjects) and £50 for each subsequent year, covering all tutorial classes, but not including instruction in infectious fevers, pharmacy, and vaccination. Terms for qualified practitioners may be ascertained from the Medical Secretary to the School, from whom particulars may also be obtained of the five entrance scholarships and a number of prizes and research scholarships offered for competition among the students and newly qualified men.

**University College Hospital.**—Open to men and a limited number of women students who have passed a recognised examination in anatomy and physiology. The preliminary and intermediate studies may be carried out at University College or any other recognised medical school. Whole-time directors of medical and surgical units, with the aid of whole-time assistants, carry out the systematic training of the student in the principles of medicine and surgery, while the practical side is mainly dealt with by the honorary staff of the hospital. Scholarships, exhibitions, and prizes to the value of over £1,000 are offered for competition every year. Composition fees for the final M.B., B.S. course of the University of London and for the medical education required by the Examining Board in England and the Society of Apothecaries, for the course required for the third examination, 112 guineas, or in two instalments of 70 and 45 guineas. The Dental School, Great Portland Street, W. (formerly the National Dental Hospital), has been recently reorganised and equipped with the most modern appliances for the teaching of dental surgery. The complete dental curriculum can be carried out at University College and University College Hospital. Particulars of the fees for the dental curriculum can be obtained from the Sub-Dean for dental students at the Dental School, Great Portland Street.

**Westminster Hospital Medical School.**—Westminster Hospital was the first institution of its kind in London which was founded by the voluntary contributions of the public. It affords relief to upwards of 2,600 in-patients and about 25,000 out-patients annually. There is a Students' Clubs Union, the subscription for membership of which is included in the general fees. The annual composition fee is 35 guineas. Women students are admitted. Five Entrance Scholarships are offered for competition amongst students entering in October and two for those entering in May. The Dean, from whom further particulars can be obtained, is Dr. A. S. Woodwark, M.D., F.R.C.P., C.M.G., C.B.E.

